# Predicting circRNA–disease associations with shared units and multi-channel attention mechanisms

**DOI:** 10.1093/bioinformatics/btaf088

**Published:** 2025-03-05

**Authors:** Xue Zhang, Quan Zou, Mengting Niu, Chunyu Wang

**Affiliations:** School of Computer Science and Technology, Harbin Institute of Technology, Harbin, Heilongjiang 150000, China; Institute of Fundamental and Frontier Sciences, University of Electronic Science and Technology of China, Chengdu, Sichuan 610000, China; Institute of Fundamental and Frontier Sciences, University of Electronic Science and Technology of China, Chengdu, Sichuan 610000, China; Yangtze Delta Region Institute (Quzhou), University of Electronic Science and Technology of China, Quzhou, Zhejiang 324000, China; Institute of Fundamental and Frontier Sciences, University of Electronic Science and Technology of China, Chengdu, Sichuan 610000, China; School of Electronic and Communication Engineering, Shenzhen Polytechnic University, Shenzhen, Guangdong 518055, China; School of Computer Science and Technology, Harbin Institute of Technology, Harbin, Heilongjiang 150000, China

## Abstract

**Motivation:**

Circular RNAs (circRNAs) have been identified as key players in the progression of several diseases; however, their roles have not yet been determined because of the high financial burden of biological studies. This highlights the urgent need to develop efficient computational models that can predict circRNA–disease associations, offering an alternative approach to overcome the limitations of expensive experimental studies. Although multi-view learning methods have been widely adopted, most approaches fail to fully exploit the latent information across views, while simultaneously overlooking the fact that different views contribute to varying degrees of significance.

**Results:**

This study presents a method that combines multi-view shared units and multichannel attention mechanisms to predict circRNA–disease associations (MSMCDA). MSMCDA first constructs similarity and meta-path networks for circRNAs and diseases by introducing shared units to facilitate interactive learning across distinct network features. Subsequently, multichannel attention mechanisms were used to optimize the weights within similarity networks. Finally, contrastive learning strengthened the similarity features. Experiments on five public datasets demonstrated that MSMCDA significantly outperformed other baseline methods. Additionally, case studies on colorectal cancer, gastric cancer, and nonsmall cell lung cancer confirmed the effectiveness of MSMCDA in uncovering new associations.

**Availability and implementation:**

The source code and data are available at https://github.com/zhangxue2115/MSMCDA.git.

## 1 Introduction

The Circular RNAs (circRNAs) are single-stranded RNA molecules characterized by their closed-loop structure, which lacks both 5' cap and 3' poly-A tail (Hwang *et al.* 2024, Liu *et al.* 2024a,b,c,d). Emerging evidence suggests that circRNA contributes to numerous biological processes including regulating gene expression as miRNA sponges and facilitating gene transcription in the nucleus through protein interactions (Panda 2018, Guo *et al.* 2023). The stability conferred by their circular form against exonuclease activity highlights their potential as reliable biomarkers and therapeutic targets for various diseases (Zhang *et al.* 2018, Manayalan *et al.* 2019, Ai *et al.* 2023), such as circSLC8A1, which inhibits tumour growth in prostate cancer by sequestering miR-21 (Wang *et al.* 2021a,b). Similarly, circSLC25A16 promotes glycolysis in nonsmall cell lung cancer (NSCLC) (Shangguan *et al.* 2020, Liu *et al.* 2024a,b,c,d). These findings emphasize the pivotal role of circRNAs in disease progression (Hsiao *et al.* 2017, Hong *et al.* 2020), indicating their potential for advancing drug development and precision medicine.

Despite some circRNA–disease associations have been identified through wet-lab experiments, the expense and time demands have prevented the exploration of many potential associations (Sun *et al.* 2022, Qiao *et al.* 2024). Researchers have developed various reliable computational models to address this issue (Wang *et al.* 2021a,b, Ren *et al.* 2024). The classification of these models includes three groups: network propagation models, traditional machine learning models, and deep learning models.

Network-based methods predict new associations by constructing heterogeneous networks using known associations and calculating their similarities (Yang *et al.* 2024, Zhu *et al.* 2024). In the KATZHCDA model, a heterogeneous network was formed using three different circRNA similarity measures, and the KATZ method was applied to predict associations (Fan *et al.* 2018). IBNPKATZ initially constructs the similarity matrix between circRNAs and diseases, and subsequently uses bipartite network projection along with the KATZ method to compute the predicted scores (Zhao *et al.* 2019). CD-LNLP designs linear neighbourhood similarities for circRNAs and diseases, and subsequently uses label propagation(LP) approach to predict association probabilities (Zhang *et al.* 2019). Ge *et al.* utilizes Locality-Constrained Linear Coding to rebuild the cosine similarity of circRNAs and diseases. Afterward, the reconstructed circRNA similarity network and disease similarity network, along with the semantic similarities of circRNAs and diseases, were used in LP method to obtain the score matrix (Ge *et al.* 2020). Overall, although similarity-based models can effectively predict circRNA–disease associations, they often overemphasize similarity scores.

Traditional machine learning-based models predict associations by training classifiers using manually extracted features (Manavalan *et al.* 2019, Yin *et al.* 2024). The GBDTCDA method uses a gradient-boosting decision tree to analyse features from a similarity network (Lei and Fang 2019). RNMFLP model first updates the association matrix through matrix multiplication to integrate known associations and subsequently utilizes robust nonnegative matrix factorization along with a label propagation method (Peng *et al.* 2022). The MLCDA model applies principal component analysis to fused similarity features to extract key attributes and calculate association scores using inductive matrix completion (Wang *et al.* 2022). Although machine learning models significantly enhance the efficiency of association prediction, their performance relies heavily on extracted features. Similar to similarity-based models, these methods largely rely on various similarity measures. However, not all circRNAs and diseases possess suitable similarity attributes. For example, when calculating disease semantic similarity, some diseases are not included in certain databases, which results in a similarity value of zero, severely affecting the model's performance.

Deep learning-based methods excel at the extraction of latent features (Liu *et al.* 2024a,b,c,d, Wang *et al.* 2024, Zhang *et al.* 2024a,b). Moreover, because multiple views offer richer information than a single view, they are frequently used in deep learning (Zhu *et al.* 2023, Yang *et al.* 2024). The GEHGAN model constructs similarity features along with features derived from a random walk method that uses skip-and-stay strategies. Next, the features are integrated through a multi-head graph attention, and a MLP is used for prediction (Wang and Lu 2024). By incorporating both the local and global neighbour features of circRNAs and diseases, GGAECDA utilizes a graph autoencoder for joint training to predict associations (Li *et al.* 2022). CLCDA establishes three distinct association views to derive features through constraints among these views. It then reconstructs the network by fusing these features, and uses a Graph Autoencoder module to predict potential associations (Wang *et al.* 2023). Although these methods achieve notable performance, they do not effectively leverage the latent features across various views. Moreover, they do not account for the varying importance of the different views.

To overcome these issues, we introduced MSMCDA, a new approach that leverages shared units and attention mechanisms for circRNA–disease association prediction. First, MSMCDA constructs similarity and meta-path networks for circRNAs and diseases and designs shared units to facilitate the exchange and fusion of information between similarity and meta-path networks. We then used attention mechanisms to merge features across multiple similarity networks. Finally, contrastive learning enhances the features from the similarity networks, and an MLP classifier is used to predict the associations.

**Figure 1.**
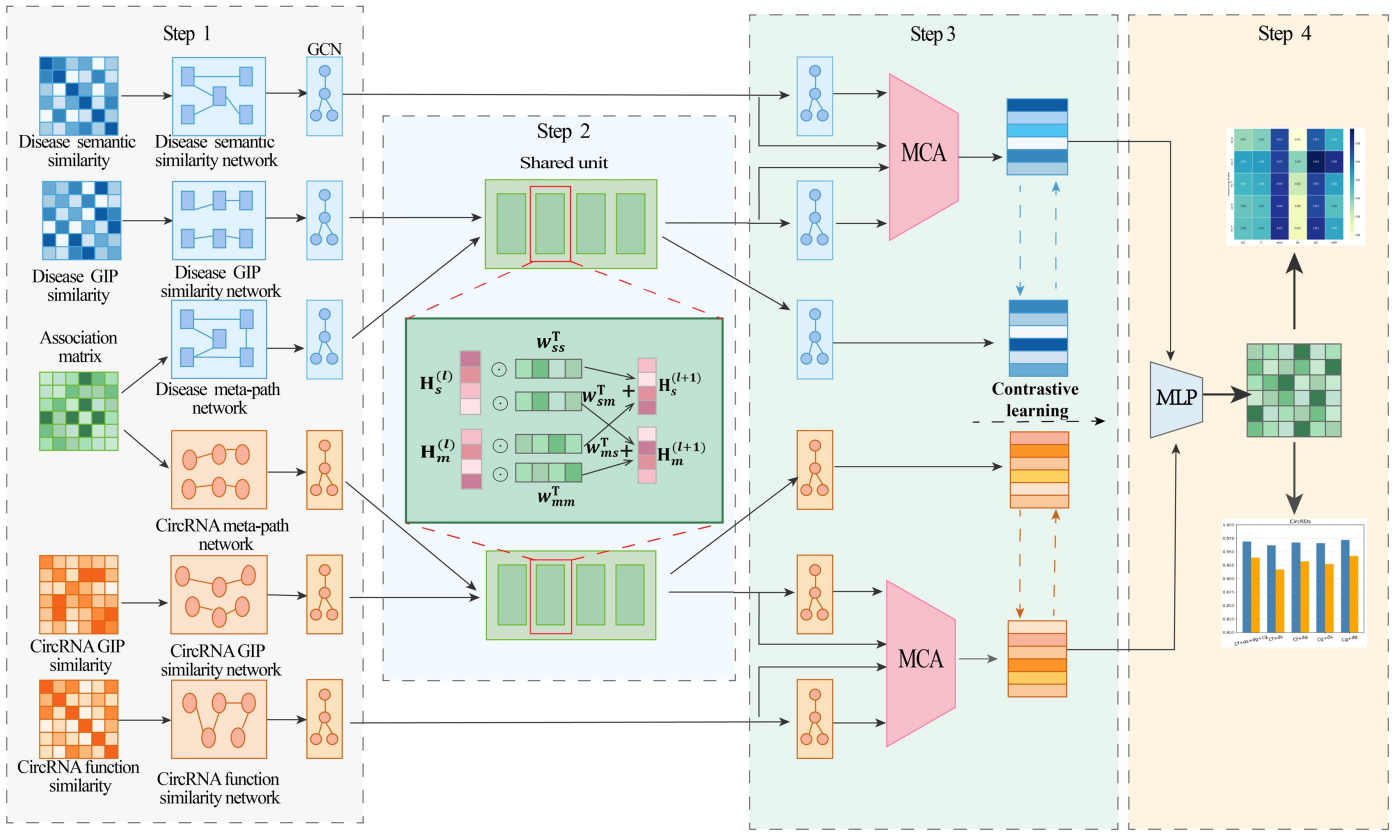
The overall workflow of MSMCDA. In step 1, MSMCDA constructs the similarity networks and meta-path networks for circRNAs and diseases. In step 2, we design shared units to facilitate the exchange and fusion of information between similarity networks and meta-path networks. In step 3, MSMCDA use attention mechanisms to integrate features from different similarity networks, and use contrastive learning to enhancing feature representation. In step 4, MSMCDA predicts the potential circRNA–disease associations with MLP classifier.

Figure 1 presents the workflow of MSMCDA. The key contributions of this study are as follows:

1. MSMCDA introduces shared units that facilitate feature interaction across views, enabling complementary integration during the fusion process, and capturing potential cross-view information.
2. MSMCDA uses attention mechanisms to assign different significance levels to the features of various similarity networks.
3. MSMCDA introduces a contrastive learning approach to fully leverage complementary information across views, which enhances feature by aligning similar features and distinguishing dissimilar ones, thereby improving the model's ability to capture meaningful information.
4. We evaluated the effectiveness of MSMCDA through comprehensive experiments on five widely used public datasets for circRNA–disease associations. The results reveal that MSMCDA achieves superior performance than state-of-the-art baseline methods.

## 2 Materials and methods

### 2.1 Datasets

The Five benchmark datasets were tested to evaluate the performance of MSMCDA: CircR2Disease, CircR2Diseasev2.0, CircRNADisease, Circ2Disease, and CircRDS. CircR2Disease, CircRDS, CircRNADisease, and Circ2Disease data were obtained from a previous study (Peng *et al.* 2022). They included 650 (585 circRNAs and 88 diseases), 310 (298 circRNAs and 33 diseases), 270 (249 circRNAs and 59 diseases), and 868 (748 circRNAs and 108 diseases) validated associations, respectively. CircR2Diseasev2.0 was sourced from publicly available databases (Fan *et al.* 2022). We eliminated nonhuman entries and standardized the names of the different circRNAs and diseases according to the information provided. Ultimately, CircR2Diseasev2.0, included 1642 associations involving 1229 circRNAs and 141 diseases. The detailed characteristics of the datasets are summarized in Table 1.

**Table 1.** Details of circRNA–disease association data.

Datasets	circRNAs	Diseases	Associations
CircR2Disease	585	88	650
CircR2Diseasev2.0	1229	141	1642
CircRNADisease	249	59	270
Circ2Disease	298	33	310
CircRDS	748	108	868

The associations in each dataset are expressed using an adjacency matrix $A\in R^{N_{c}\times N_{d}}.$ A matrix element of 1 represents a connection between The associations in each dataset are expressed using an adjacency matrix$A\in R^{N_{c}\times N_{d}}.$ A matrix element of 1 represents a connection between a circRNA and a disease, whereas 0 indicates the absence of connection. Here,$N_{c}$and$N_{d}$refer to the numbers of circRNAs and diseases, respectively.

### 2.2 CircRNAs and diseases similarity networks construction

Similarity was measured using two methods: semantic and GIP similarities were applied to diseases, whereas functional and GIP similarities were used for the circRNAs.

#### 2.2.1 Construction of disease similarity networks

We retrieved the Disease Ontology identity (DOID) for each disease from the disease ontology dataset (Kibbe *et al.* 2015) for disease semantic similarity, and calculated the similarity using the Dosim function provided in the R language package (Li *et al.* 2011). In the disease ontology framework, each disease is represented by a directed acyclic graph (DAG). The formula in the Dosim function is defined as:

(1) SDdi,dj=∑t∈Ndi∩Ndj Sdit+Sdjt∑t∈Ndi Sdit+∑t∈Ndj Sdjt

In the DAG of $d_{i}$, $N_{d_{i}}$ refers to ancestral diseases and $S_{d_{i}}\left(t\right)$ quantifies the semantic contribution of diseasetto disease $d_{i}$, defined as follows:

(2) {Sdit=1otherwiseSditmax=0.5*Sdit′∣t′∈childrenoftift≠di

Given the sparsity of disease semantic similarity data, which limits the comprehensive characterization of disease features, GIP similarity was incorporated to enrich the similarity feature information. A kernel function was used to calculate the GIP similarity between diseases i and j based on their corresponding columns in the association matrix as follows:

(3) GDdi,dj=exp⁡-ηd∥Adi-Adj∥2

where $d_{i}$ and $d_{j}$ refer to the ith and jth columns of the association matrix, with $\eta_{d}$ indicating the normalized kernel bandwidth. The formula for calculation is as follows:

(4) ηd=1/1Nd∑i=1Nd ∥Adi∥

where$N_{d}$denotes the number of columns.

The edge weights in $G_{ds}$, the disease semantic similarity network, are determined by $SD\left(d_{i},d_{j}\right)$, and those in $G_{dg}$, the disease GIP similarity network, are determined by $GD\left(d_{i},d_{j}\right)$.

#### 2.2.2 Construction of circRNA similarity networks

The functional similarity of circRNAs suggests that those with similar functions tend to be linked to similar diseases. The formula used to compute the functional similarity of circRNA $c_{i}$ and $c_{j}$ is as follows:

(5) FCci,cj=∑d∈Qi SDd,Qj+∑d∈Qj SDd,QiQi+Qj

Where $Q_{i}$ and $Q_{j}$ denote the sets of diseases linked to circRNA $c_{i}$ and circRNA $c_{j}$, respectively.

Similar to diseases, circRNAs also use GIP similarity to enrich similarity information.

(6) GCci,cj=exp⁡-ηc∥Aci-Acj∥2

 

(7) ηc=1/1Nc∑i=1Nc ∥Aci∥

where $c_{i}$ and $c_{j}$ correspond to the ith and jth rows of the association matrix, respectively, and $N_{c}$denotes the number of rows.

Similar to the disease networks, the circRNA functional similarity network $G_{cf}$ uses edge weights defined by FCci,cj and the GIP similarity network $G_{cg}$ uses $GC\left(c_{i},c_{j}\right)$.

#### 2.2.3 Construction of meta-path networks

The meta-path comprehensively represents the structural connections between circRNAs and diseases, enabling the capture of detailed structural information within the association network (Zhang *et al.* 2023). To use this information, MSMCDA developed meta-path networks for circRNAs and diseases based on different meta-paths.

We defined two meta-paths: $m_{1}$ = CDC for circRNAs and $m_{2}$ = DCD for diseases. The respective meta-path networks were built using these meta-paths. The circRNA network was formulated as follows:

(8) Am1=A×AT

where *A* denotes the circRNA–disease association network and $A^{T}$ is the transpose of association network *A*.

In parallel, the meta-path network $m_{2}$ for diseases is given by:

(9) Am2=AT×A

The edge weights in $G_{m1}$ and $G_{m2}$ are determined from their corresponding $A_{m1}$ and $A_{m2}$ values, respectively.

### 2.3 Feature extraction from multi-view similarity networks

Given the effectiveness of GCNs in embedding node features within graph structures (Yan *et al.* 2023, Zhang *et al.* 2024a,b), we used them to extract similarity and meta-path features from constructed circRNA and disease networks. This approach enables the efficient integration and enhancement of node features across networks. After one layer of GCN convolution, the updated features of circRNAs were calculated as follows:

(10) HScl+1=σD∼c-12S∼cD∼c-12HSclWScl

where ${\tilde{S}}_{c}=S_{c}+I$, $S_{c}$ is the adjacency matrix of the circRNA similarity network with an added identity matrixI, $\tilde{D_{c}}$ is the corresponding degree matrix, $W_{S_{c}}$ represents the learnable parameters, σ is the activation function, and $H_{S_{c}}$denotes the circRNA node features in layer l.

Similarly, for disease similarity network $S_{d}$, the updated embeddings at layer l+1 are given by:

(11) HSdl+1=σD∼d-12S∼dD∼d-12HSdlWSdl

where ${\tilde{S}}_{d}=S_{d}+I$ represents the sum of the adjacency matrix of the disease similarity network and an identity matrix; $\tilde{D_{d}}$ is the degree matrix, and $H_{S_{d}}$is the disease node features in layer l.

The feature learning process for meta-path networks follows the same approach. For a meta-path network $A_{m_{n}}\left(n\in \{1,2\}\right)$, the node features at layer l+1 are given by:

(12) HAmnl+1=σD∼-12A∼mnD∼-12AmnlWAmnl,n∈1,2

where ${\tilde{A}}_{m_{n}}$ is the adjacency matrix of the meta-path network with an added identity matrix, $\tilde{D}$ is its degree matrix, and $H_{A_{m_{n}}}^{\left(l\right)}$ represents the node features in layer l.

#### 2.3.1 Shared unit

To enhance feature alignment and promote cross-view interaction between similarity information and meta-path information in circRNA and disease networks, this study introduces a novel sharing unit comprising four linear operation modules, each designed to facilitate mutual learning across views.

Taking circRNA as an example, the GIP similarity network features and meta-path network features were initially processed by the first linear module. Subsequently, four trainable weights adaptively determine the relative importance of each input feature in each linear operation.

(13) Hsl+1=wssT⊙Hml+wsmT⊙Hsl

 

(14) Hml+1=wmmT⊙Hsl+wmsT⊙Hml

where $H_{s}^{\left(l+1\right)}$and $H_{m}^{\left(l+1\right)}$ denote the updated features of the similarity and meta-path views, respectively. The operator ⊙ denotes element-wise multiplication, $w_{ss}^{T},$  $w_{sm}^{T}$, $w_{mm}^{T}$ and $w_{ms}^{T}$are trainable parameters.

The sharing unit serves as a bridging mechanism between views, facilitating cross-view feature interactions to iteratively refine the input representations. In this study, the sharing unit was strategically positioned between the first and second GCN layers in both GIP similarity and meta-path views, enabling the effective integration of information at an intermediate representation level.

#### 2.3.2 Attention mechanism

Considering that the node features extracted from distinct similarity views affect the results differently (Tang *et al.* 2021, Wei *et al.* 2021), we introduced multichannel attention mechanisms to adaptively adjust the importance of each view.

First, the importance coefficient $\alpha_{c}$ for the circRNA similarity view is calculated as follows:

(15) HscGCN=GAPcHfc1,Hfc2,Hgc1,Hgc2

 

(16) αc=δW2σW1HscGCN

where ${\mathrm{GAP}}_{c}$ denotes global average pooling, $W_{1}$ and $W_{2}$ represent fully connected layers, the ReLU activation function is denoted by σ, and the sigmoid function is represented by δ.

The adjusted circRNA similarity features are computed as follows:

(17) HscAtt=CNNcσαc⋅HscGCN

where *σ* (·) is ReLU activation function and ${\mathrm{CNN}}_{c}$ denotes a 2D convolutional neural network used to merge multiple similarity views.

The importance coefficients and adjusted disease similarity features are computed as:

(18) HsdGCN=GAPdHsd1,Hsd2,Hgd1,Hgd2

 

(19) αd=δW2σW1HsdGCN

 

(20) HsdAtt=CNNdσαd⋅HsdGCN

Where ${\mathrm{GAP}}_{c}$ denotes global average pooling, $W_{1}$ and $W_{2}$ represent fully connected layers, the ReLU activation function is denoted by σ, and the sigmoid function is represented by δ.

#### 2.3.3 Contrastive learning

Contrastive learning is used to maximize the similarity within positive samples and minimise it between negative samples during the training process to improve the feature representation (Sheng *et al.* 2023, Liu *et al.* 2024a,b,c,d). Positive samples refer to features of the same entity derived from different views, whereas negative samples refer to features of different entities across views.

The contrastive learning loss for circRNAs is defined as:

(21) LC=-log⁡esimcisn,cimpesimcisn,cimp+∑j∈Nesimcisn,cjmp

where *N* represents the set of negative samples *i* ≠ *j*, and sim(*a*, *b*) denotes the cosine similarity function.

(22) sima,b=-aTb∥a∥⋅∥b∥

The contrastive learning loss for diseases follows the same equation:

(23) Ld=-log⁡esimdisn,dimpesimdisn,dimp+∑j∈Nesimdisn,djmp

#### 2.3.4 Final decoder

In the prediction phase, circRNAs and disease features were combined using element-wise multiplication and then passed into fully connected layers. Subsequently, a sigmoid activation function is used to output the final prediction score.

(24) y^ij=SigmoidFNNHci⊙Hdj

where $H_{c_{i}}$ and $H_{d_{j}}$ denote the embeddings for circRNAs $c_{i}$ and diseases $d_{j}$,respectively. The operation ⊙ denotes the element-wise multiplication, and the FNN is fully connected layers. The sigmoid function is the activation function.

To optimize the entire model, this study used the binary cross-entropy loss, defined as:

(25) LB=-∑i,j∈Y-∪Y+yijlog⁡y^ij+1-yijlog⁡1-y^ij

where *Y*+ and *Y*− indicate positive and negative samples in the training data, respectively. When $(i,j)\in Y^{+}$, the actual label is set to 1; otherwise, it is set to 0.

The final loss function L for MSMCDA, which integrates the two loss functions of the contrastive learning strategy, is given by:

(26) L=LB+Lc+Ld#

#### 2.3.5 Implementation details

For training, MSMCDA used the Adam optimizer (Kinga and Adam 2015) and set the learning rate to 0.001. Both the circRNA and disease embeddings were set to 128 dimensions. The model achieved optimal performance after 128 training epochs.

## 3 Results and discussion

### 3.1 Experimental setup and evaluation metrics

The experimental dataset for MSMCDA comprised equal numbers of positive and negative samples. We used 5-fold cross-validation (5-CV) to evaluate MSMCDA and compared it with baseline methods across Circ2disease, CircRNAdisease, CircRDS, CircR2disease, and CircR2diseasev2.0. In the 5-CV process, the entire set of circRNA–disease associations was split into five equal parts. In each cross-validation, one part acted as the test set and the remaining four were used for training.

This study evaluated the model performance using the area under the receiver operating characteristic curve (AUC), area under the precision-recall curve(AUPR), accuracy, F1-score, recall, and precision (Qian *et al.* 2023, Zou *et al.* 2023). To minimise potential bias in 5-CV, the mean performance across all folds was reported as the final metric.

To verify the effectiveness of MSMCDA, experiments were conducted on five datasets with varying sample sizes. The results of MSMCDA on the five datasets are presented in Table 2. The results showed that MSMCDA performed well across all the datasets, highlighting its robustness and generalizability to datasets of different scales.

**Table 2.** MSMCDA's performance across various datasets.

	ACC	F1	Recall	Pre	AUC	AUPR
Circ2disease	0.883	0.875	0.950	0.818	0.933	0.882
CircRNAdisease	0.95	0.95	0.97	0.93	0.988	0.968
CircR2disease	0.923	0.925	0.925	0.897	0.976	0.962
CircR2v2.0disease	0.932	0.933	0.944	0.922	0.968	0.952
CircRDS	0.93	0.933	0.97	0.9	0.972	0.942

### 3.2 Comparison with other methods

We evaluated MSMCDA by comparing its performance with six state-of-the-art approaches: MDGF-MCEC (Wu *et al.* 2022), Bi-SGTAR (Li *et al.* 2024), GraphCDA (Dai *et al.* 2022), DMFCDA (Lu *et al.* 2021), GMNN2CD (Niu *et al.* 2022), AMHMDA (Ning *et al.* 2023). For comparison purposes, experiments were performed using the same five datasets. The AUC and AUPR results of different methods on the five datasets are shown in Table 3. All results are provided in Supplementary Table S1 of the Supplementary File.

**Table 3.** The AUC and AUPR results of different methods on five datasets.

		AMHMDA	MDGF-MCEC	Bi-SGTAR	GMNN2CD	GraphCDA	DMFCDA	Ours
CircR2disease	AUC	0.757	0.927	0.780	0.954	0.931	0.499	0.976
	AUPR	0.714	0.929	0.722	0.604	0.893	0.530	0.964
CircR2diseasev2.0	AUC	0.909	0.936	0.755	0.858	0.916	0.882	0.968
	AUPR	0.89	0.942	0.799	0.838	0.874	0.846	0.952
CircRNAdisease	AUC	0.734	0.961	0.933	0.936	0.949	0.829	0.988
	AUPR	0.665	0.957	0.927	0.944	0.929	0.857	0.968
Cir2disease	AUC	0.753	0.908	0.883	0.887	0.929	0.680	0.947
	AUPR	0.696	0.908	0.868	0.869	0.893	0.767	0.908
CircRDs	AUC	0.847	0.933	0.782	0.974	0.925	0.718	0.972
	AUPR	0.804	0.939	0.792	0.792	0.883	0.711	0.942

MSMCDA showed strong predictive capability for CDA, achieving an AUC of 0.976 for CircR2disease in 5-CV, exceeding the second-best score by 0.022. In addition, MSMCDA achieved the highest AUC values across all the datasets, demonstrating excellent predictive capabilities. Notably, the AMHMDA model was designed for miRNA datasets; because circRNA data are relatively sparse, it does not perform well on sparse data. Models using multi-view approaches, such as MDGF-MCEC and GraphCDA, significantly outperform single-view models such as Bi-SGTAR and DMFCDA, indicating that multi-view methods can provide additional information. Among these, MDGF-MCEC and GraphCDA ranked just below MSMCDA in terms of performance. Although these methods also use multi-view methods, they lack shared learning between views, which limits their ability to fully exploit the cross-view information. Overall, our method demonstrated robust performance in predicting the CDA.

### 3.3 Parameter sensitivity analysis

Hyperparameters are essential factors that determine the performance of the MSMCDA model. This study mainly concentrates on three parameters: number of linear modules, embedding dimension, and learning rate. We analysed the impact of these parameters on the results and compared the evaluation metrics.

**Figure 2.**
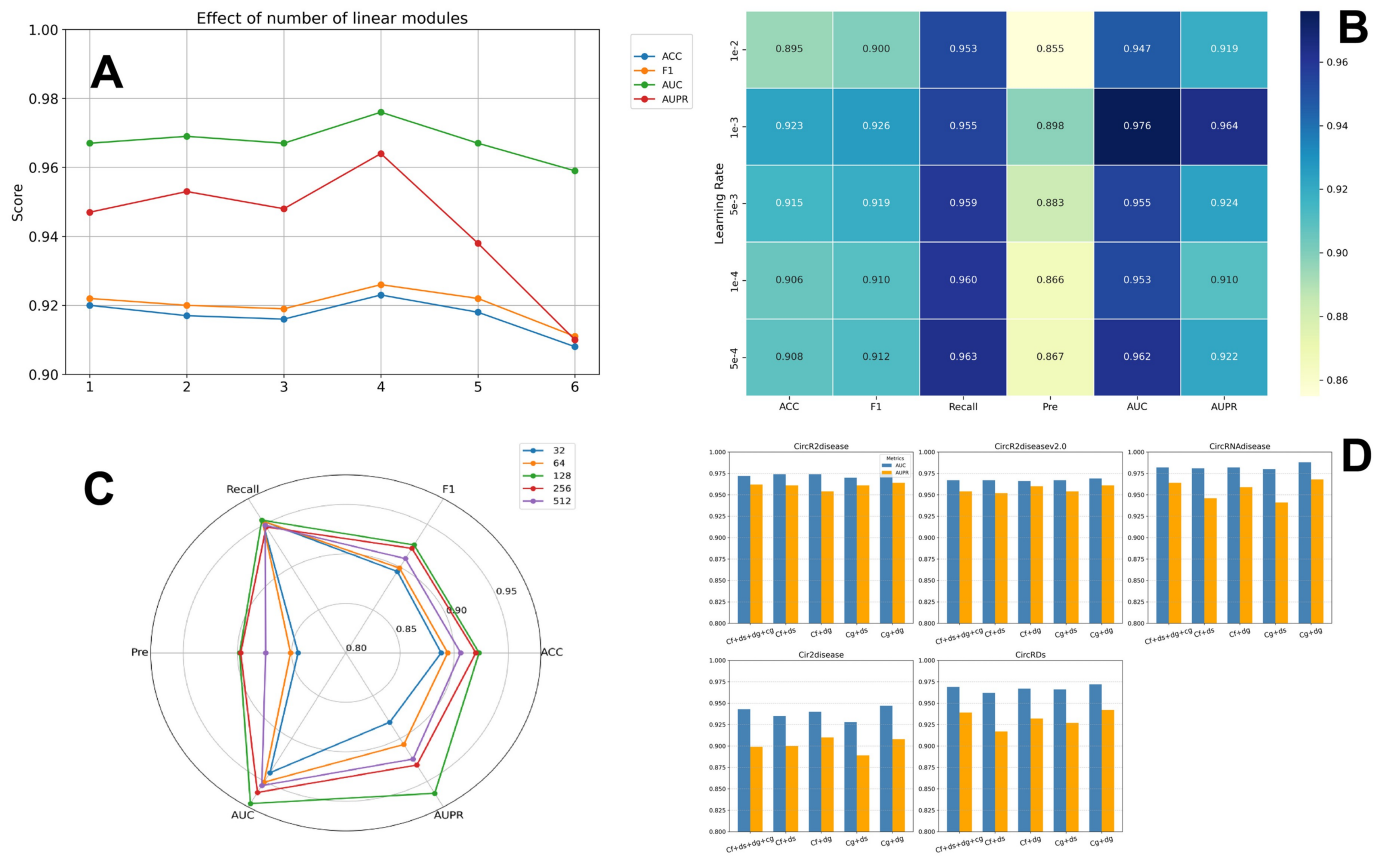
(A) Parameter analysis for number of linear modules. (B) Parameter analysis for learning rate. (C) Parameter analysis for the feature embedding dimension. (D) The performance of MSMCDA with different shared view combinations.

The first parameter is the number of linear modules in each shared unit. Here, l represents the number of linear modules. Figure 2A illustrates the experimental results under varying l values. When l > 4, model performance begins to decline. This study chose l = 4 as the ideal number of linear modules.

The second parameter was the feature embedding dimension. The embedding size determined the dimensions of associated learnable-parameter matrices. As shown in Fig. 2C, the evaluation metrics were recorded for embedding dimensions 32, 64, 128, and 256. The results indicated that the model performance varied with the embedding size, achieving the best outcome in 128 dimensions. Thus, we selected 128 as the feature dimension for all nodes to achieve and maintain optimal model performance.

The final parameter was the learning rate, which controlled the speed at which the model adjusted its parameters during the training. An improperly set learning rate, whether too high or too low, may significantly degrade model performance. Therefore, MSMCDA tests the learning rates of 1e-2, 1e-3, 5e-3, 1e-4, and 5e-4 to identify the optimal value. Figure 2B illustrates that the model performed best at a learning rate of 1e-3, which was consequently adopted in this study.

### 3.4 The impact of shared view combinations

To thoroughly investigate the effect of different views after passing through the shared unit, we designed five schemes with various view combinations and evaluated their performances. The performance results for these view combinations are shown in Fig. 2D. Among the five combinations, the results of sharing the GIP similarity and meta-path views outperformed the others, showing the best performance. This is likely because both views are derived from relational associations and exhibit similar structures, thus enabling them to capture latent information more effectively. Consequently, GIP similarity and meta-path views were selected for sharing in this study.

### 3.5 Ablation study

Ablation experiments were conducted on multiple datasets to analyse the contributions of each module. Three model variants were designed for comparison: (i) MSMCDA-noatten, which eliminates attention mechanisms and assigns equal importance to all views, (ii) MSMCDA-noCL, which removes contrastive learning and uses aggregated similarity features for prediction, and (iii) MSMCDA-noshare, which excludes shared units and applies two GCN layers directly to the raw input features without any sharing.

The AUC and AUPR results of Ablation experiments are shown in Table 4. All results are provided in Supplementary Table S2 of the Supplementary File. As shown in Table 4, MSMCDA outperformed the other variants on the five datasets. MSMCDA-noshare performed the worst, highlighting the importance of the shared units, which allow different view features to interact, learn more latent information, and improve the model's performance. Furthermore, the attention mechanisms demonstrated a notable impact on the results, suggesting that features from different layers contribute to varying degrees of importance. Finally, the performance of MSMCDA-noCL highlights the role of contrastive learning in enhancing feature representation and improving model performance.

**Table 4.** Ablation experiment results of MSMCDA on five datasets.

Dataset	Methods	AUC	AUPR
CircR2Disease	MSMCDA	0.976	0.964
	MSMCDA-noatten	0.97	0.938
	MSMCDA-noshare	0.925	0.882
	MSMCDA-noCL	0.969	0.933
CircR2DiseaseV2.0	MSMCDA	0.969	0.961
	MSMCDA-noatten	0.967	0.953
	MSMCDA-noshare	0.896	0.893
	MSMCDA-noCL	0.966	0.933
CircRNADisease	MSMCDA	0.988	0.968
	MSMCDA-noatten	0.982	0.961
	MSMCDA-noshare	0.965	0.944
	MSMCDA-noCL	0.983	0.952
Circ2Disease	MSMCDA	0.947	0.908
	MSMCDA-noatten	0.927	0.86
	MSMCDA-noshare	0.859	0.80
	MSMCDA-noCL	0.92	0.873
CircRDS	MSMCDA	0.972	0.942
	MSMCDA-noatten	0.969	0.934
	MSMCDA-noshare	0.928	0.895
	MSMCDA-noCL	0.97	0.939

### 3.6 Case study

To assess the model’s ability to predict unknown associations, we removed the known associations of three diseases: colorectal cancer, gastric cancer, and NSCLC, and retrained the model on the remaining dataset. The model was then used to predict the scores for unknown associations between these diseases and the circRNAs. Finally, the top ten circRNAs were validated through searches of PubMed and other literatures.

Colorectal cancer is a major cancer of the digestive system with high rates of occurrence and death. However, it is highly treatable if it is detected at an early stage. Circ-ZNF609 influences colorectal cancer migration by modulating glioma-associated oncogene 1 expression via miRNA-150 (Wu *et al.* 2018). Hsa_circRNA_000167 facilitates colorectal cancer development by modulating the miR-326/EPHB3 axis.

Gastric cancer is a prevalent malignancy characterized by high incidence and low early detection rates. The lack of obvious symptoms in the initial phase lowers the chances of effective treatment in the later stages. As shown in Table 5, seven of the top ten ranked circRNAs were identified. For example, hsa_circ_0000993 may inhibit the spread of gastric cancer by interacting with miR-214-5p (Zhong *et al.* 2018), and circ _0081143 suppresses the progression of DR-resistant gastric cancer by regulating miR-129-2-3p, highlighting its potential as a therapeutic target for DR-resistant gastric cancer (Ou *et al.* 2022).

**Table 5.** The top 10 associations identified by MSMCDA for gastric cancer, colorectal cancer, nonsmall cell lung cancer.

Disease	Rank	circRNA	PMID
Gastric cancer	1	hsa_circ_0000993	30215537
	2	circ_0081143	30733646
	3	hsa_circ_0076704	36934850
	4	hsa_circ_0067103	Not available
	5	hsa_circ_0034398	[33]
	6	circTHBS1	35338119
	7	circCER	33262782
	8	hsa_circRNA_101981	Not available
	9	hsa_circ_0076906	Not available
	10	circRNA_100395	33841580
Colorectal cancer	1	hsa_circ_0000615	30570857
	2	hsa_circRNA_000167	[34]
	3	circCTDP1	39143504
	4	hsa_circ_0001162	34841662
	5	circSCARB1	Not available
	6	circEXOC7	Not available
	7	hsa_circ_0087378	34488530
	8	hsa_circ_0000092	39296099
	9	hsa_circ_0001839	Not available
	10	Circ-UBR1	34787049
Nonsmall cell lung cancer	1	hsa_circRNA_102049	30873868
	2	circMYLK	32753964
	3	CircRNA-0044073	30864721
	4	hsa_circ_0072088	32524752
	5	circOSBPL10	31409903
	6	hsa_circ_0141539	32550825
	7	circ-UBE2D2	35196197
	8	hsa_circRNA_014213	Not available
	9	hsa_circ_0001206	31198063
	10	circ-102166	Not available

NSCLC constitutes >80% of all lung cancer diagnoses. The absence of effective diagnostic methods often delays diagnosis until advanced stages, leading to poor treatment outcomes and reduced survival rates. Hsa_circRNA_102049 enhances the growth and spread of NSCLC cells by regulating miR-520f, making it a potential candidate for biomarker (Cui *et al.* 2019). CircRNA MYLK promotes the proliferation of NSCLC cells by binding to miR-195-5p (Xiong *et al.* 2020).

## 4 Conclusions

Extensive research has indicated that circRNAs are crucial in various aspects of disease, including diagnosis, treatment, and prognosis. This study presents MSMCDA, a model that leverages shared units and multichannel attention mechanisms to predict circRNA–disease associations. MSMCDA uses shared units to enable the viewing of interactions between similarity and meta-path views. It then uses multichannel attention mechanisms to integrate meta-path networks and similarity networks, followed by a contrastive learning strategy to enhance feature representations. Experiments on datasets from five databases revealed that MSMCDA outperformed the six advanced methods. Case studies further demonstrated MSMCDA's effectiveness in identifying potential circRNA–disease associations, highlighting its practical utility. Despite achieving promising results in predicting circRNA–disease associations, MSMCDA still has limitations. One of the primary limitations is the limited number of meta-paths used in the model. The inclusion of additional meta-paths could provide more comprehensive information, improving the accuracy of association predictions. Furthermore, the model could be enhanced by incorporating more diverse biological data, which may help in capturing more complex relationships between circRNAs and diseases. In future research, we aim to expand the meta-paths used in the model and integrate additional biological data sources to further enhance its predictive performance.

## Supplementary Material

btaf088_Supplementary_Data

## Data Availability

The data underlying this article are available in CircR2Disease database, CircRDS database, CircRNADisease database, and Circ2Disease database at http://doi.org/10.1093/bib/bbac155, CircR2Diseasev2.0 database at http://bioinfo.snnu.edu.cn/CircR2Disease_v2.0.
