# Supplementary material for: Predicting circRNA–disease associations with shared units and multi-channel attention mechanisms
Source: Bioinformatics. 2025 Mar 5;41(3):btaf088. doi: 10.1093/bioinformatics/btaf088 (PMC11919450; doi:10.1093/bioinformatics/btaf088)
Supplement: btaf088_Supplementary_Data [file btaf088_supplementary_data.docx]

| Model | ACC | F1 | Recall | Pre | AUC | AUPR |
| --- | --- | --- | --- | --- | --- | --- |
| Ours | 0.923 | 0.925 | 0.925 | 0.897 | 0.976 | 0.964 |
| AMHMDA | 0.712 | 0.701 | 0.677 | 0.727 | 0.757 | 0.714 |
| MDGF-MCEC | 0.845 | 0.842 | 0.831 | 0.855 | 0.927 | 0.929 |
| Bi-SGTAR | 0.573 | 0.638 | 0.823 | 0.587 | 0.780 | 0.722 |
| GMNN2CD | 0.518 | 0.466 | 0.450 | 0.982 | 0.954 | 0.604 |
| GraphCDA | 0.931 | 0.932 | 0.951 | 0.913 | 0.931 | 0.893 |
| DMFCDA | 0.582 | 0.453 | 0.377 | 0.629 | 0.499 | 0.530 |

**Supplementary Table 1(a). The results of different methods on** **CircR2disease**

| Model | ACC | F1 | Recall | Pre | AUC | AUPR |
| --- | --- | --- | --- | --- | --- | --- |
| Ours | 0.932 | 0.933 | 0.944 | 0.922 | 0.968 | 0.952 |
| AMHMDA | 0.827 | 0.833 | 0.866 | 0.814 | 0.909 | 0.890 |
| MDGF-MCEC | 0.863 | 0.862 | 0.862 | 0.864 | 0.936 | 0.942 |
| Bi-SGTAR | 0.683 | 0.607 | 0.489 | 0.799 | 0.755 | 0.799 |
| GMNN2CD | 0.55 | 0.20 | 0.1 | 0.92 | 0.858 | 0.838 |
| GraphCDA | 0.916 | 0.917 | 0.937 | 0.899 | 0.916 | 0.874 |
| DMFCDA | 0.852 | 0.855 | 0.867 | 0.843 | 0.882 | 0.846 |

**Supplementary Table 1(b). The results of different methods on** **CircR2diseasev2.0**

| Model | ACC | F1 | Recall | Pre | AUC | AUPR |
| --- | --- | --- | --- | --- | --- | --- |
| Ours | 0.95 | 0.95 | 0.97 | 0.93 | 0.988 | 0.968 |
| AMHMDA | 0.654 | 0.643 | 0.652 | 0.657 | 0.734 | 0.665 |
| MDGF-MCEC | 0.882 | 0.878 | 0.852 | 0.907 | 0.961 | 0.957 |
| Bi-SGTAR | 0.687 | 0.559 | 0.397 | 0.946 | 0.933 | 0.927 |
| GMNN2CD | 0.571 | 0.243 | 0.142 | 0.96 | 0.936 | 0.944 |
| GraphCDA | 0.948 | 0.948 | 0.939 | 0.957 | 0.949 | 0.929 |
| DMFCDA | 0.819 | 0.798 | 0.752 | 0.872 | 0.829 | 0.857 |

**Supplementary Table 1(c). The results of different methods on circRNAdisease**

| Model | ACC | F1 | Recall | Pre | AUC | AUPR |
| --- | --- | --- | --- | --- | --- | --- |
| Ours | 0.897 | 0.90 | 0.938 | 0.865 | 0.947 | 0.908 |
| AMHMDA | 0.672 | 0.677 | 0.698 | 0.690 | 0.753 | 0.696 |
| MDGF-MCEC | 0.785 | 0.777 | 0.755 | 0.804 | 0.908 | 0.908 |
| Bi-SGTAR | 0.857 | 0.865 | 0.911 | 0.823 | 0.883 | 0.868 |
| graphCDA | 0.929 | 0.931 | 0.948 | 0.915 | 0.929 | 0.893 |
| DMFCDA | 0.726 | 0.874 | 0.533 | 0.66 | 0.680 | 0.767 |
| GMNN2CD | 0.529 | 0.109 | 0.059 | 0.8 | 0.887 | 0.869 |

**Supplementary Table 1(d). The results of different methods on Cir2disease**

| Model | ACC | F1 | Recall | Pre | AUC | AUPR |
| --- | --- | --- | --- | --- | --- | --- |
| Ours | 0.93 | 0.933 | 0.97 | 0.9 | 0.972 | 0.942 |
| AMHMDA | 0.752 | 0.752 | 0.776 | 0.745 | 0.847 | 0.804 |
| MDGF-MCEC | 0.857 | 0.850 | 0.818 | 0.886 | 0.933 | 0.939 |
| Bi-SGTAR | 0.804 | 0.805 | 0.809 | 0.801 | 0.782 | 0.792 |
| GMNN2CD | 0.974 | 0.310 | 0.184 | 1.0 | 0.974 | 0.792 |
| graphCDA | 0.925 | 0.927 | 0.953 | 0.902 | 0.925 | 0.883 |
| DMFCDA | 0.708 | 0.656 | 0.561 | 0.792 | 0.718 | 0.711 |

**Supplementary Table 1(e). The results of different methods on circRDs**

|  | ACC | F1 | Recall | Pre | AUC | AUPR |
| --- | --- | --- | --- | --- | --- | --- |
| MSMCDA | 0.922 | 0.925 | 0.959 | 0.894 | 0.976 | 0.964 |
| MSMCDA-noatten | 0.925 | 0.927 | 0.960 | 0.897 | 0.970 | 0.938 |
| MSMCDA-noshare | 0.858 | 0.868 | 0.934 | 0.812 | 0.925 | 0.882 |
| MSMCDA-noCL | 0.926 | 0.929 | 0.929 | 0.928 | 0.969 | 0.933 |

**Supplementary Table 2(a). Ablation experiment results of MSMCDA on CircR2disease**

|  | ACC | F1 | Recall | Pre | AUC | AUPR |
| --- | --- | --- | --- | --- | --- | --- |
| MSMCDA | 0.897 | 0.9 | 0.938 | 0.865 | 0.947 | 0.908 |
| MSMCDA-noatten | 0.883 | 0.889 | 0.942 | 0.844 | 0.927 | 0.86 |
| MSMCDA-noshare | 0.806 | 0.812 | 0.842 | 0.787 | 0.859 | 0.80 |
| MSMCDA-noCL | 0.872 | 0.879 | 0.939 | 0.830 | 0.92 | 0.873 |

**Supplementary Table 2(b). Ablation experiment results of MSMCDA on Cir2disease**

|  | ACC | F1 | Recall | Pre | AUC | AUPR |
| --- | --- | --- | --- | --- | --- | --- |
| MSMCDA | 0.95 | 0.95 | 0.97 | 0.93 | 0.988 | 0.968 |
| MSMCDA -noatten | 0.938 | 0.938 | 0.968 | 0.912 | 0.982 | 0.961 |
| MSMCDA -noshare | 0.903 | 0.907 | 0.943 | 0.876 | 0.965 | 0.944 |
| MSMCDA -noCL | 0.93 | 0.932 | 0.967 | 0.90 | 0.983 | 0.952 |

**Supplementary Table 2(c). Ablation experiment results of MSMCDA on circRNAdisease**

|  | ACC | F1 | Recall | Pre | AUC | AUPR |
| --- | --- | --- | --- | --- | --- | --- |
| MSMCDA | 0.93 | 0.933 | 0.97 | 0.9 | 0.972 | 0.942 |
| MSMCDA-noatten | 0.93 | 0.933 | 0.967 | 0.901 | 0.969 | 0.934 |
| MSMCDA-noshare | 0.866 | 0.875 | 0.938 | 0.822 | 0.928 | 0.895 |
| MSMCDA -noCL | 0.922 | 0.924 | 0.96 | 0.892 | 0.97 | 0.939 |

**Supplementary Table 2(d). Ablation experiment results of MSMCDA on circRDs**

|  | ACC | F1 | Recall | Pre | AUC | AUPR |
| --- | --- | --- | --- | --- | --- | --- |
| MSMCDA | 0.931 | 0.932 | 0.949 | 0.916 | 0.969 | 0.961 |
| MSMCDA-noatten | 0.927 | 0.928 | 0.943 | 0.915 | 0.967 | 0.953 |
| MSMCDA-noshare | 0.799 | 0.804 | 0.758 | 0.854 | 0.896 | 0.893 |
| MSMCDA-noCL | 0.93 | 0.933 | 0.967 | 0.90 | 0.966 | 0.933 |

**Supplementary Table 2(e). Ablation experiment results of MSMCDA on CircR2diseasev2.0**
